# Comparative sequence analysis of leucine-rich repeats (LRRs) within vertebrate toll-like receptors

**DOI:** 10.1186/1471-2164-8-124

**Published:** 2007-05-21

**Authors:** Norio Matsushima, Takanori Tanaka, Purevjav Enkhbayar, Tomoko Mikami, Masae Taga, Keiko Yamada, Yoshio Kuroki

**Affiliations:** 1 School of Health Sciences, Sapporo Medical University, Hokkaido 060-8556, Japan; 2 RIKEN Genomic Sciences Center, Yokohama, Kanagawa 230-0045, Japan; 3 Faculty of Biology, National University of Mongolia, Ulaanbaatar-210646/377, Mongolia; 4 Department of Nursing, Sapporo City University, Sapporo, Hokkaido 060-0011, Japan; 5 Department of Biochemistry, School of Medicine, Sapporo Medical University, Hokkaido 060-8556, Japan

## Abstract

**Background:**

Toll-like receptors (TLRs) play a central role in innate immunity. TLRs are membrane glycoproteins and contain leucine rich repeat (LRR) motif in the ectodomain. TLRs recognize and respond to molecules such as lipopolysaccharide, peptidoglycan, flagellin, and RNA from bacteria or viruses. The LRR domains in TLRs have been inferred to be responsible for molecular recognition. All LRRs include the highly conserved segment, LxxLxLxxNxL, in which "L" is Leu, Ile, Val, or Phe and "N" is Asn, Thr, Ser, or Cys and "x" is any amino acid. There are seven classes of LRRs including "typical" ("***T***") and "bacterial" ("***S***"). All known domain structures adopt an arc or horseshoe shape. Vertebrate TLRs form six major families. The repeat numbers of LRRs and their "phasing" in TLRs differ with isoforms and species; they are aligned differently in various databases. We identified and aligned LRRs in TLRs by a new method described here.

**Results:**

The new method utilizes known LRR structures to recognize and align new LRR motifs in TLRs and incorporates multiple sequence alignments and secondary structure predictions. TLRs from thirty-four vertebrate were analyzed. The repeat numbers of the LRRs ranges from 16 to 28. The LRRs found in TLRs frequently consists of LxxLxLxxNxLxxLxxxxF/LxxLxx ("***T***") and sometimes short motifs including LxxLxLxxNxLxxLPx(x)LPxx ("**S**"). The *TLR7 *family (TLR7, TLR8, and TLR9) contain 27 LRRs. The LRRs at the N-terminal part have a super-motif of ***STT ***with about 80 residues. The super-repeat is represented by ***STTSTTSTT ***or ***_TTSTTSTT***. The LRRs in TLRs form one or two horseshoe domains and are mostly flanked by two cysteine clusters including two or four cysteine residue.

**Conclusion:**

Each of the six major TLR families is characterized by their constituent LRR motifs, their repeat numbers, and their patterns of cysteine clusters. The central parts of the *TLR1 *and *TLR7 *families and of TLR4 have more irregular or longer LRR motifs. These central parts are inferred to play a key role in the structure and/or function of their TLRs. Furthermore, the super-repeat in the *TLR7 *family suggests strongly that "bacterial" and "typical" LRRs evolved from a common precursor.

## Background

**Figure 1:**
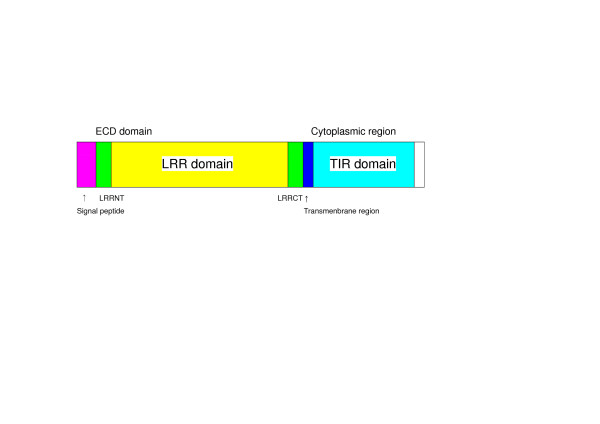
Structural organization of vertebrate TLRs. Mangenta is signal peptide sequence. Green is LRRNT (the cysteine clusters on the N-terminal side of LRRs) and LRRCT (the cysteine clusters on the C-terminal side of LRRs). Yellow is LRR domain. Blue is transmembrane region. Light blue is TIR domain.

Toll-like receptors (TLRs) play a central role in innate immunity [1-3]. TLRs are type I integral membrane glycoproteins consisting of leucine rich repeat (LRR) motif in the ectodomain (ECD), and cytoplamic signaling domains known as Toll IL-receptor (TIR) domains, joined by a single trans membrane helix (Figure 1). They recognize and respond to a variety of components derived from pathogenic or commensal microorganisms principally bacteria and viruses. These molecules include lipids such as lipopolysaccharide (LPS) from Gram-negative bacteria and peptidoglycan fragments from bacterial cell walls, proteins such as flagellin and nucleic acids such as single-stranded and double-stranded RNA and unmethylated CpG DNA from bacteria or viruses. The ECDs including LRRs have been inferred to recognize directly various ligands. The TLR family counts 10 members in human and 12 in mice and Takifugu rubripes. Six major families of vertebrate TLRs have been proposed in a molecular dendrogram [4].

Leucine-rich repeat (LRR)-containing domains are present in over 6000 proteins listed in PFAM, PRINTS, SMART, and InterPro data bases [5-8]. All LRR repeats can be divided into a highly conserved segment (HCS) and a variable segment (VS). The HCS part consists of an eleven residue stretch, LxxLxLxxNxL, or a tweleve residue stretch, LxxLxLxxCxxL, in which "L" is Leu, Ile, Val, or Phe, "N" is Asn, Thr, Ser, or Cys, and "C" is Cys, Ser or Asn [7,9]. Seven classes of LRRs have been proposed, characterized by different lengths and consensus sequences of the VS part of repeats [9,10]. They are "RI-like", "CC", "bacterial", "SDS22-like", "plant specific", "typical", and "TpLRR". Each subfamily of small leucine-rich repeat proteoglycan (SLRP) has LRRs from more than one of the seven classes [8,11]. The structures of twenty-two different proteins that contain LRRs are available [12-51]. They include TLR3 and CD14 [48-50]. The LRR domains in all known structures adopt an arc shape. Most of the known LRR structures have a cap, which shields the hydrophobic core of the first LRR at the N-terminus or the last LRR at the C-terminus. In extracellular proteins or extracellular domains, these caps frequently consist of cysteine clusters including two or four cysteine residues [8,9].

The indicated repeat number of LRRs and its "phasing" (that is, what segment or residue corresponds to the beginning of a repeating unit) in individual TLRs are different among the databases (or researchers) and species. This difference reflects the irregularity of LRR motifs in TLRs. Over one hundred complete TLRs are available. Several methods of protein secondary structure predictions such as Proteus and SSPro4.0 show a correspondence of about 75% [52-54]. For the identification of LRRs we propose a new method, which uses the known structures of several LRRs, multiple sequence alignments and secondary structure predictions of TLRs. This new method indicates that each of the six recognized TLR families can be characterized by its LRR motifs, their repeat number and the motifs of two cysteine clusters flanking the LRRs. The actual repeat number of LRRs is generally larger than those reported in the databases. The present analysis leads to the hypothesis that all the LRRs in TLRs form one or two horseshoe domains.

## Results

### A new method for the identification of LRRs within TLRs

#### LRR known structures

All of the LRR domains in one protein form a single continuous structure and adopt an arc or horseshoe shape. On the inner, concave face there is a stack of parallel β-strands and on the outer, convex face there are a variety of secondary structures such as α-helix, 3_10_-helix, polyproline II helix, or a tandem arrangement of β-turns [8,55]. The HCS part of all the LRRs consists of LxxLxLxxNxL or LxxLxLxxCxxL,, as noted, in which "L" is Leu, Ile, Val, or Phe, "N" is Asn, Thr, Ser, or Cys, and "C" is Cys, Ser or Asn [7,9]. The short β-strands are mostly formed by three residues at positions 3 through 5 in the HCS part. In most LRR proteins the β-strands on the concave face and (mostly) helical elements on the convex face are connected by short loops or β-turns. Four leucine residues at positions 1, 4, 6 and 11 participate in the hydrophobic core in LRR arcs. Similarly, conserved hydrophobic residues in the VS parts of the seven LRR classes participate in the hydrophobic core. The side chains of asparagine at position 9 form hydrogen bonds in the loop structure [6].

Structural alignments of the known LRR structures reveal that the LRR motif is surprisingly variable (Table 1). The lengths of LRRs range from 20 to 43 residues. Leucines at positions 1, 4, 6 and 11 of the HCS part are sometimes replaced by Met, Ala, or Cys, as seen in TLR3 [49,50], Internalin A (Inl-A) [26], and Internalin B (Inl-B) [22-24]. Leucines at positions 1 and 11 are also occupied by relatively hydrophilic residues such as Gly, Thr, Asn and Tyr. Furthermore, asparagine at position 9 is occupied by hydrophobic residues such as Val, Leu and Ile. It is clear that many LRRs do not keep the complete HCS pattern and are irregular. Eighteen of the 22 known structures contain irregular LRRs. Most of the irregular HCSs can be classified into four groups; LxxLxLxx(**L/I/M)**xL, LxxLxLxx(**R**/**K**/**E)**xL, LxxLxLxx(N/S/T/**A)**xx, and LxxLxLxx**x**x**x **in which residues in boldface are irregular. Also there are rare examples, **xV**xxLxLxxNxL and **P**xxLxLxxNxL in follicle-stimulating hormone receptor (FSHr), and LxxLx**G**xxS/PxI in Inl-C and DLC1 (Table 1). Furthermore, an irregular LRR with (L/x)xx(L/A)xCxx(**L/R)**xLxxVPxxIPxx, which belongs to the "bacterial" motif, is frequently observed at the first LRR (LRR1) at the N-terminus of the LRR domain (Table 1).

**Table 1:** Irregular LRR motifs observed in the known structures of LRR-containing proteins

Protein^a^	N^b^	Irregular LRRs			PDB
		Position	Amino acid sequence^c^		
			**LxxLxLxxNxL**		
TLR3	25	LRR1	**H**EVADCSH**L**KL	TQVPDDLPTN	1ZIW
		LRR9	LFGLFLNN**V**QL	GPSLTEKLCLELANTS	/2A0Z
		LRR13	VRYLNLKRSF**T**	KQSISLASLPKIDDFSFQWLKC	
		LRR15	LKYLSLSNSF**T**	SLRTLTNETFVSLAHSP	
		LRR18	IFEIYLSYNK**Y**	LQLTRNSFALVPS	
		LRR19	LQRLMLRR**V**AL	KNVDSSPSPFQPLRN	
CD14	11	LRR1	AADVELYG**G**G**R**	SLEYLLKRVDTEADLGQFTDIIKSLS	1WWL
		LRR2	LKRLTVRA**A**RI	PSRILFGALRVLGISG	
		LRR3	LQELTLEN**L**EV	TGTAPPPLLEATGPD	
		LRR4	LNILNLRN**V**SW	ATRDAWLAELQQWLKPG	
		LRR5	LKVLSIAQ**A**H**S**	LNFSCEQVRVFPA	
		LRR7	LQVLALRN**A**GM	ETPSGVCSALAAARVQ	
Slit	6	LRR1	**G**TTVDCTG**R**GL	KEIPRDIPLH	1W8A
Lingo-1	14	LRR8	LIVLRLRH**L**NI	NAIRDYSFKRLYR	2ID5
		LRR9	LKVLEISH**W**P**Y**	LDTMTPNCLYGLN	
Decorin	12	LRR1	LRVVQCSD**L**GL	EKVPKDLPPD	1XCD
Biglycan	12	LRR1	LRVVQCSD**L**GL	KAVPKEISPD	2FT3
FSHr	10	LRR6	LQKVLLDI**Q**D**N**	INIHTIERNSFVGLSFE	1XWD
		LRR7	**S**VILWLNKNGI	QEIHNCAFNGTQ	
		LRR9	**P**VILDISRTRI	HSLPSYGLEN	
Inl-A	16	LRR1	VTTLQADR**L**GI	KSIDGLEYLNN	1O6V
Inl-C	7	LRR1	VQNFN**G**DNSNI	QSLAGMQFFTN	1XEU
		LRR7	VNWIDLTG**Q**KC	VNEPVKYQPEL	
U2snRNPA'	5	LRR1	DRELDLRG**Y**KI	PVIRNLGATLDQ	1A9N
RabGGTα	5	LRR1	VRVLHLAH**K**DL	TVLCHLEQLLL	1DCE
DLC1	6	LRR1	**K**VELH**G**MI**P**PI	EKMDATLSTLKA	1DS9
TAP	4	LRR1	**Q**QALDLKG**L**R**S**	DPDLVAQNIDVVLNRRSCMAATLRII	1FT8
				EENIPE	
PGIP	10	LRR1	VNNLDLSG**L**NL	PKPYPIPSSLANLPYL	1OGQ
YoPM	16	LRR1	AHELELNN**L**GL	SSLPELPPH	1G9U
		LRR16	VEDLRMNS**E**RV	DPYEFAHETTDKLEDDVFE	
hRI	16	LRR15	LEQLVLYD**I**YW	SEEMEDRLQALEKDKP	1Z7X
pRI	17	LRR1	W**_**NLDIHC**E**QL	SDARWTELPLQQ	2BNH
RanGAP	10	LRR2	LEIAEFSD**I**F**T**	GRVKDEIPEALRLLLQALLKCPK	1YRG
cTmod	5	LRR1	LEEVNLNN**I**M**N**	IPVPTLKACAEALKTNTY	1IO0
		LRR2	VKKFSIVGTR**S**	NDPVAFALAEMLKVNNT	
		LRR4	LIELRIDN**Q**S**Q**	PLGNNVEMEIANMLEKNTT	
		LRR5	LLKFG**Y**HFTQ**Q**	PRLRASNAMMNNNDLVRK	
ceTmod	5	LRR1	LKEVNINN**M**KR	VSKERIRSLIEAACNSKH	1PGV
		LRR4	IVEFKADN**Q**R**Q**	SVLGNQVEMDMMMAIEENES	
		LRR5	LLRVGISF**A**S**M**	EARHRVSEALERNYERVRL	
Skp2	11	LRR1	WQTLDLTG**K**NL	HPDVTGRLLSQG	1FQV
		LRR4	LQNLSLWF**L**RL	SDPIVNTLAKNSN	/2ASS
		LRR7	ITQLNLSG**Y**R**K**	NLQKSDLSTLVRRCPN	
		LRR10	LKTLQVFG**I**V**P**	DGTLQLLKEA	

^a^FSHr, follicle-stimulating hormone receptor; Inl-A, internalin A; Inl -C, internalin C; U2snRNPA', spliceosomal U2A' protein; RabGGTα, rab geranylgeranyltransferase α-subunit; DLC-1, *Chlamydomonas *outer arm dynein light chain 1; TAP, mRNA export factor; PGIP, polygalacturonase-inhibiting protein; RI, ribonuclease inhibitor, RanGAP, GTPase-activating protein; Tmod, tropomodulin; Skp2, S-phase kinase-associated protein 2; h, human, p, pig; ce, Caenorhabditis elegans: ^b ^N; the repeat number of LRRs identified in individual known LRR structures.^c ^HCS, highly conserved segment of LRR; VS, variable segment of LRR. Residues in boldface cause irregular motif.

#### Multiple sequence alignment

**Figure 2:**
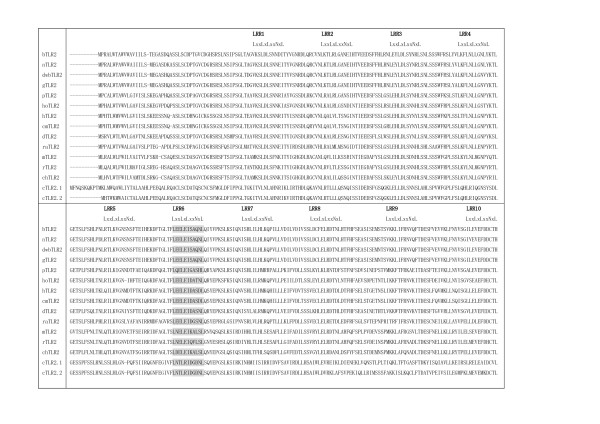
The multiple sequence alignment of LRRs within mammalian TLR2 from 14 species. bTLR2   [Q95LA9], nTLR2 [Q2V897], dwbTLR2 [Q2PZH4], gTLR2 [ABI31733], pTLR2 [Q59HI8], hoTLR2   [AAR08196], hTLR2 [O60603], cmTLR [Q95M53], dTLR2 [Q689D1], raTLR2 [AAM50059], mTLR2 [Q9QUN7],   rTLR2 [Q6YGU2], chTLR2 [Q9R1F8], cTLR2.1 [Q9DD78], cTLR2.2 [Q9DGB6].  Abbreviations: b, Bovine;   n, nilgai; dwb, domestic water buffalo; g, goat;  p, pig; ho, horse; h, human; cm, Cynomolgus   monkey; d, dog ; ra, rabbit; m, mouse; r, rat; ch,  Chinese hamster; c, chicken.   This panel   shows the sequences from the N-termini to LRR10.

**Figure 3:**
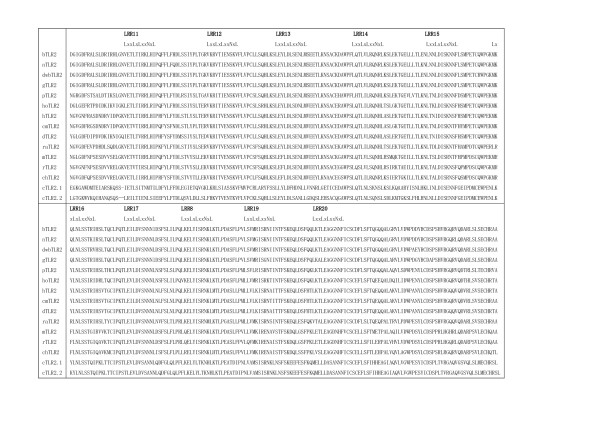
The multiple sequence alignment of LRRs within mammalian TLR2 from 14 species. This panel continued from Figure 2 shows the sequences from LRR11 to the C-termini.

Mammalian TLR2 contains 20 LRRs, as described later. The PFAM program detects only 5–7 of the 20 LRRs, while the InterPro database (20 August, 2006) counts 13 in chicken, 14 in human, Cynomolgus monkey, dog and Chinese hamster, and 18 in bovine (Table 2). Figures 2 and 3 show the multiple sequence alignment of the LRR domain in mammalian TLR2 from 14 species. The sixth LRR (LRR6) shows canonical and irregular LRRs whose HCS parts consist of LxxLxIxx(S/T/N)xL and LxxLxIxx(**Q/D)**xL or LxxLxIxx**L**xL, respectively. The VS part is "typical". Both canonical and irregular LRR are also seen in LRR9 and LRR10. Furthermore, the HCS part of LRR4 shows LxxLxLxxNxY in which position 11 is tyrosine. This pattern was recognized in the known structures of TLR3 and lingo-1 (Table 1). The pairwise sequence identities are >35%. Thus, all LRRs in TLR2s from the 14 species can be reasonably regarded as an LRR motif.

**Table 2:** The repeat number of LRRs and its flanking cysteine clusters in vertebrate TLRs

Famliy^a^	Protein	Species^b^	N_L_^c^	LRRNT^d^	LRRCT^d^	N_B_^e^
*TLR1*	TLR1	h, m, p	20(8–9)	No	*CxCx*_25_*Cx*_20_*C*	1
	"	t	21	*CxCx*_29_*Cx*_20_*C*	*CxCx*_29_*Cx*_20_*C*	1
	TLR2	h, m, p, b, r, d, ra, g, ho, dwb, ha, cm, n	20(14–18)	*Cx*_5_*C*	*CxCx*_24_*Cx*_20_*C*	1
	TLR2.1	c	20(13)	*Cx*_2_*Cx*_5_*CxC*	*CxCx*_24_*Cx*_20_*C*	1
	TLR2.2	c	20(13)	*Cx*_2_*Cx*_5_*CxC*	*CxCx*_24_*Cx*_20_*C*	1
	TLR2	t	20	*Cx*_2_*Cx*_5_*CxC*	*CxCx*_25_*Cx*_20_*C*	1
	TLR2	jf	19	*Cx*_2_*Cx*_5_*CxC*	*CxCx*_25_*Cx*_20_*C*	1
	TLR2	z	21	*Cx*_3_*CxCx*_5_*CxC*	*CxCx*_24_*Cx*_20_*C*	1
	TLR6	h, m, r, p, b	20(13–14)	No	*CxCx*_25_*Cx*_20_*C*	1
	TLR10	h, p	20(12)	No	*CxCx*_25_*Cx*_20_*C*	1
	TLR14	t, z	21	*Cx*_10_*C*	*CxCx*_25_*Cx*_20_*C*	1

*TLR3*	TLR3	h, m, b, r, bu, rm, z	25(22–24)	*Cx*_8_*C*	*CxCx*_25_*Cx*_18_*C*	1
	"	t	25	*Cx*_8_*C*	*CxCx*_26_*Cx*_18_*C*	1
	"	jf	27	*Cx*_8_*C*	*CxCx*_24_*Cx*_18_*C*	1
	TLR	as	27	*Cx*_15_*C*	*CxCx*_24_*Cx*_18_*C*	1
	"	rt	27	*Cx*_15_*C*	*CxCx*_24_*Cx*_18_*C*	1
	"	go	27	*Cx*_14_*C*	*CxCx*_24_*Cx*_18_*C*	1
	TLRII	rt	27	*Cx*_6_*Cx*_17_*C*	*CxCx*_24_*Cx*_18_*C*	1

*TLR4*	TLR4	h, lg, pc, ob, or	23(21)	*Cx*_10_*C*	*CxCx*_23_*Cx*_17_*C*	1
	"	m, p, r, ch, ho, b, ab, n,	23(18–19)	*Cx*_10_*C*	*CxCx*_23_*Cx*_18_*C*	1
	"	ha, ra	23	*Cx*_10_*C*	*CxCx*_23_*Cx*_15_*C*	1
	TLR4b	z	23	*Cx*_10_*C*	*CxCx*_23_*Cx*_18_*C*	1
	TLR4	h [Q5VZ17]	16	*Cx*_10_*C*	*CxCx*_23_*Cx*_18_*C*	1
	"	d	16	No	*CxCx*_23_*C*	1

*TLR5*	TLR5	h, m, r, p, b, jhm	22(15–16)	*Cx*_11_*C*	*CxCx*_24_*Cx*_18_*C*	1
	"	t	22	*Cx*_10_*C*	*CxCx*_24_*Cx*_22_*C*	1
	"	rt	22	*Cx*_8_*C*	*Cx*_27_*C*	1
	TLRS5	t	23	*Cx*_8_*C*	*Cx*_26_*C*	1

*TLR11*	TLR11	M	25(11)	*Cx*_17_*Cx*_11_*Cx*_20_*C*	*CxCx*_24_*Cx*_19_*C*	1
	TLR12	m	24(17)	*Cx*_17_*Cx*_11_*Cx*_20_*C*	*CxCx*_24_*Cx*_19_*C*	1
	TLR13	m	27(21)	*Cx*_11_*C*	*CxCx*_24_*Cx*_16_*C*	1
	TLR21	t	27	*Cx*_10_*C*	*CxCx*_24_*Cx*_15_*C*	1
	TLR22	t	27	*Cx*_8_*C*	*CxCx*_24_*Cx*_18_*C*	1
	TLR23	t	27	*Cx*_14_*C*	*CxCx*_24_*Cx*_18_*C*	1

*TLR7*	TLR7	H, m, d	27(27–28)	*Cx*_14_*C*	*CxCx*_24_*Cx*_18_*C*	2
	"	t	27	*Cx*_12_*C*	*CxCx*_24_*Cx*_18_*C*	2
	TLR8	h, m, p	27(24–26)	*Cx*_12_*C*	*CxCx*_24_*Cx*_18_*C*	2
	"	t	27	*Cx*_13_*C*	*CxCx*_24_*Cx*_18_*C*	2
	TLR9	h, m, p, b, d, ca, ho, s, mnm	27(26)	*Cx*_9_*C*	*CxCx*_23_*Cx*_18_*C*	2
	"	t, jf, gsb	27	*Cx*_10_*C*	*CxCx*_23_*Cx*_18_*C*	2
	TLR	gp	28, 26	?	*CxCx*_24_*Cx*_18_*C*	2

*Others*	TLRa	Jl	21	*Cx*_8_*C*	*CxCx*_22_*C*_12_*C*	1
	TLRb	jl	21	*Cx*_8_*C*	*CxCx*_22_*Cx*_20_*C*	1
	TLR15	c	21	No	*CxCx*_24_*Cx*_20_*C*	2

^a^*The six families and others*. ^b^Abbreviations: ab, American bison; as, Atlantic salmon; b, bovine; c, chicken; ca, cat; ch, Chinese hamster; cm, Cynomolgus monkey; d, dog; dwd, Domestic water buffalo; g, goat; go, Goldfish; gp, green puffer; gsb, Gilthead sea bream; h, human; ha, hamster; ho, horse; jf, Japanese flounder; jhm, Japanese house mouse; jl, Japanese lamprey; lg, lowland gorilla; m, mouse; mnm, Ma's night monkey; n, Nilgai; ob, Olive baboon; or, Orangutan; p, pig; pc, Pygmy chimpanzee; r, rat; ra, rabbit; rm, Rhesus macaque; rt, Rainbow trout; s, sheep; t, Takifugu rubripes; z, Zebrafish. ^c^N_L_; the repeat number of LRRs. The number of parenthesis is N_L _reported in the Interpro datbase.^d ^LRRNT, the cysteine clusters on the N-terminal side of LRRs; LRRCT, the cysteine clusters on the C-terminal side of LRRs. ^e^N_B_; the number of horseshoe domains of the LRRs.

#### Protein secondary structure prediction

**Figure 4:**
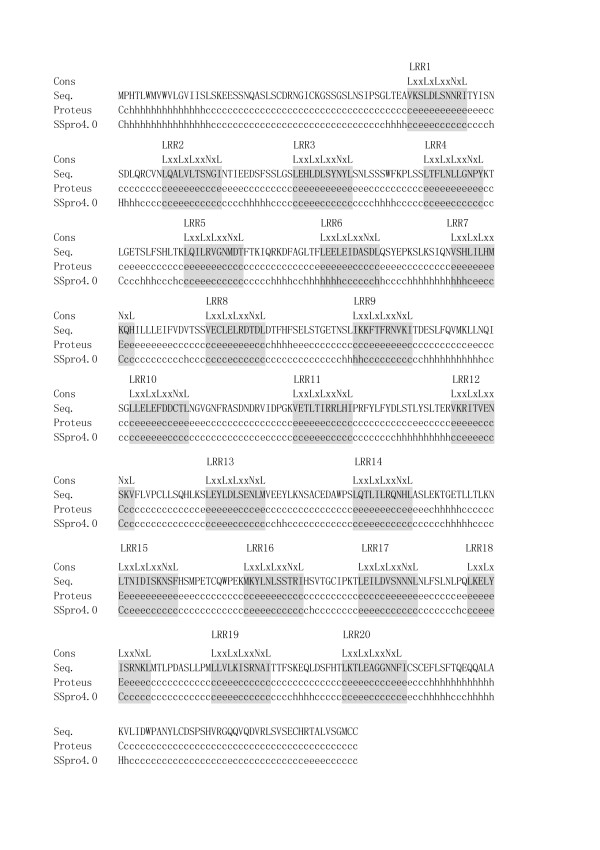
The secondary structure prediction of human TLR2 by SSpro4.0 and Proteus. The signal peptide and extracellular domain of hTLR2 [O60603] with 784 residues is shown; residues 1–588. The highly conserved segment of individual LRRs is highlighted by a shadow. Abbreviations: h, helix; c, coil; e, β-strand.

The result of the protein secondary structure prediction of human TLR2 having 20 LRRs is shown in Figure 4. Both SSpro4.0 and Proteus predict that 15 of the 20 LRRs prefer β-strands at positions 3 through 5 and/or its neighboring positions in the HCS part. They include all five irregular motifs, LRR4, LRR5, LRR7, LRR9, and LRR11. The occurrence of β-strands in LRR6 is predicted only by Proteus. However, LRR6 with the HCS part of LEELEIDASDL is clearly a canonical LRR. All twenty including LRR6 can be reasonably identified as LRR motifs.

#### The identification of LRRs within TLRs

These analyses of the known LRR structures, the multiple sequence alignments and the secondary structure predictions of TLR2 provide strong evidence that allow us to identify LRRs over an extended range of sequences and inferred structures. Taken together four steps for the identification of LRRs in each member of TLRs were used.

*Step 1*. Detection of LRRs by the PFAM program

*Step 2*. Identification of a candidate LRR that can not be recognized by PFAM.

*Step 3*: Evaluation of protein secondary structure predictions by Proteus and SSpro4.0.

*Step 4*. Determination of all LRRs in each member based on the results obtained by *Steps 1–3*.

In *Step 2*, the LRR candidates are selected using the criterion that they are longer than 18 residues and that the HCS part consisting of LxxLxLxxNxL occupies at least hydrophobic residues at positions 4 and 6. The candidate includes irregular motifs that are similar to LRRs recognized by the known structures. In case there are TLRs from many species, multiple sequence alignments are also considered for identification. In *Step 3*, the preference of β-strand in the HCS part of the LRR candidate selected by *Step 1 *and *Step 2 *was investigated. In S*tep 4*, when the candidate prefers β-strand by either Proteus or SSpro4.0 (at least in one species), it is identified as an LRR. In some cases such as LRR12 in TLR14, the initial LRR candidate was changed into another LRR based on the results of the secondary structure prediction. The crystal structure of human TLR3 [52,53] contains 25 LRRs. The present method confirmed this. In contrast, the PFAM and SMART programs predicted only 16–17 LRRs and the databases have reported 22 LRRs (Table 2).

There are two exceptions. In five mammalian TLR6s with 20 LRRs, LRR9, **P**TLLN(F/V/L)TL(N/Q)H(I/V), that contains Pro at position 1 is not predicted to have a β-strand by both prediction methods (Figure 4). However, this pattern is seen in FSHr (Table 1). Similarly, in human and pig TLR10 with 20 LRRs LRR10, GGK(A/V)YLDHNSF, is not predicted to have a β-strand by both programs (Figure 4). However, this pattern shows remarkable similarity with the sixteenth LRR (LRR16) in TLR7 and TLR8 with 27 LRRs.

### LRRs within TLRs

#### LRR motifs

**Figure 5:**
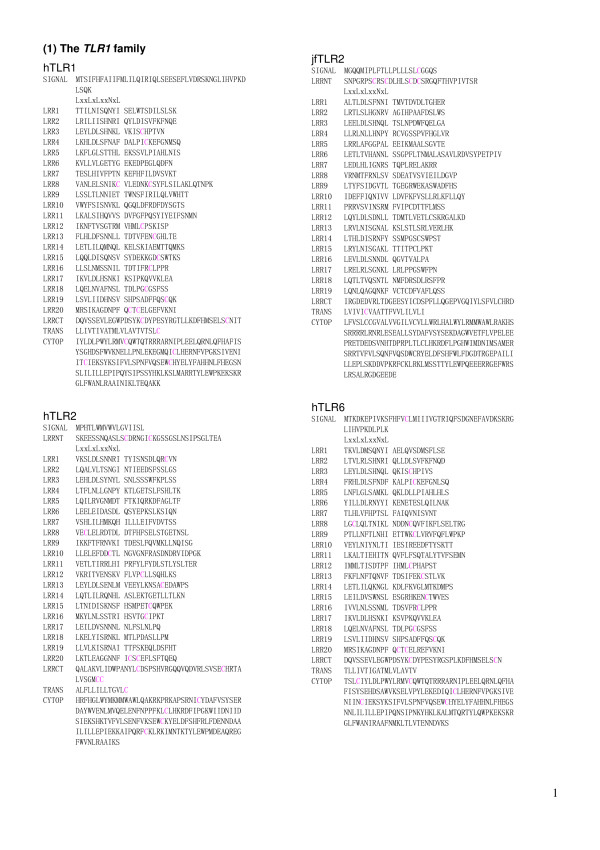
Sequence alignment of LRR domains within the six families of TLRs. (1) hTLR1   [Q15399]; hTLR2 [O60603]; hTLR6 [Q9Y2C9]; hTLR10 [Q9BXR5]; tTLR14 [Q5H726]. (2) hTLR3 [O15455];   jfTLR3 [Q76CT7]. (3) hTLR4 [O00204]; dTLR4 [Q8SQH3]. (4) hTLR5 [O60602]. (5) mTLR11 [Q6R5P0];   mTLR12 [Q6QNU9]; mTLR13[Q6R5N8]: tTLR21 [NP_001027751]; tTLR22 [Q5H723]; tTLR23 [AAW70378]; (6)   hTLR7 [Q9NYK1]; hTLR8 [Q9NR97]; hTLR9 [Q9NR96]. (7) jlTLRa [Q33E93]; cTLR15 [ABB71177].  The   complete amino acid sequences are shown for hTLR1 with 786 residues (res.), hTLR2 with 784 res,   hTLR6 with 796 res., hTLR10 with 811 res., tTLR14 with 871 res., hTLR3 with 904 res., jfTLR3   with 961 res., hTLR4 with 839 res., dTLR4 with 636 res., hTLR5 with 858 res., hTLR7 with 1049   res., hTLR8 with 1041 res., hTLR9 with 1032 res., mTLR11 with 926 res., mTLR12 with 906 res.,   mTLR13 with 991 res., tTLR21 with 965 res., tTLR22 with 950 res., tTLR23 with 941res., jlTLRa   with 813res., and cTLR15 with 868 res.,  Cysteine is highlighted in magenta. Its boldface   indicates cysteines in LRRNT or LRRCT.  Residues of missense mutation are highlighted in blue   boldface. SIGNAL, signal peptide sequence; LRRNT, the cysteine clusters on the N-terminal side   of LRRs; LRRCT, the cysteine clusters on the C-terminal side of LRRs; TRANS, transmembrane   region; CYTOP, cytoplasmic region. Abbreviations: h, human; m, mouse; t, Takifugu rubripes; c,   chicken; d, dog; jf, Japanese flounder.  This panel shows hTLR1, hTLR2, jfTLR2 and TLR6 in the   TLR1 family.

**Figure 6:**
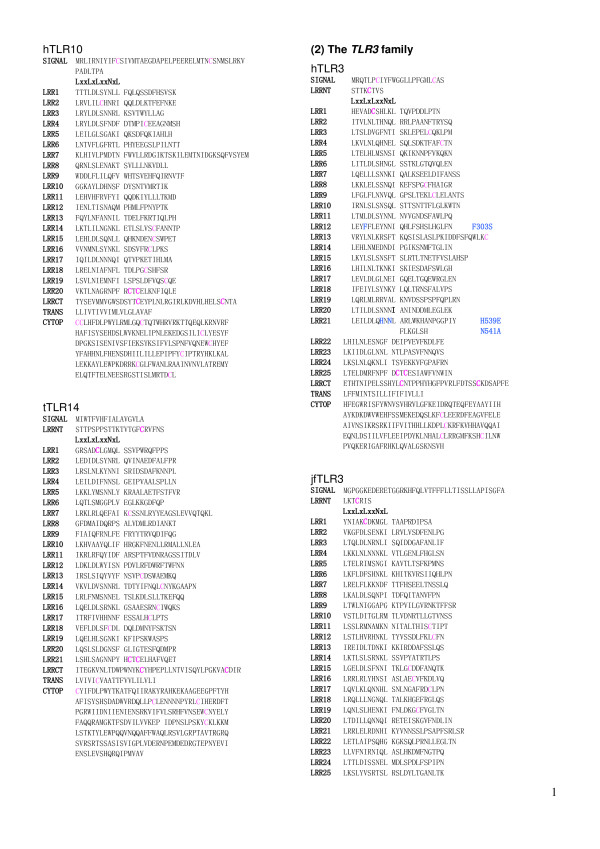
Sequence alignment of LRR domains within the six families of TLRs. This panel    continued from Figure 5 shows hTLR10 and hTLR14 in the TLR1 family, and hTLR3 and jfTLR3 in the   TLR3 family. .

**Figure 7:**
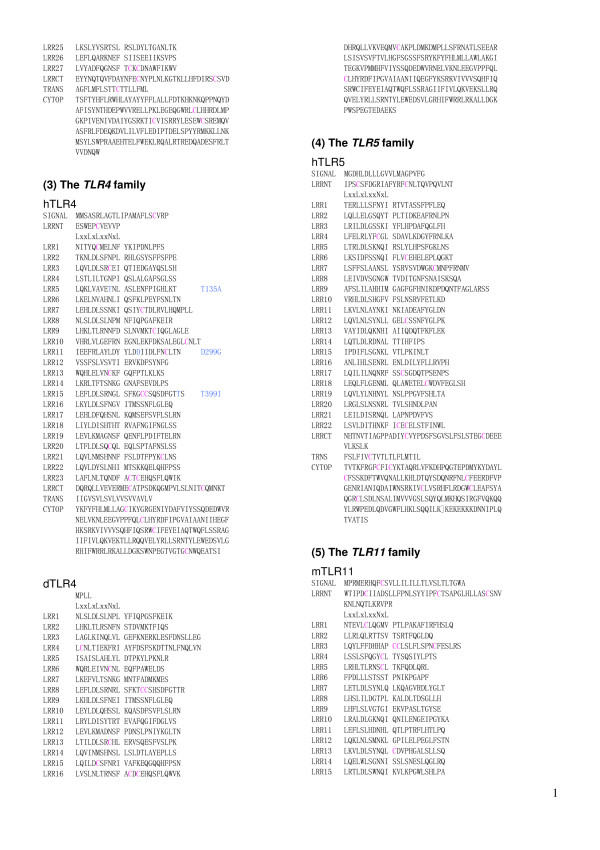
Sequence alignment of LRR domains within the six families of TLRs.  This panel   continued in Figure 6 shows jfTLR3 (from LRR25 to CYTOP in the TLR3 family, hTLR4 and dTLR4 in the TLR4 family, hTLR5in the TLR5 family, and   mtLR11 (from SIGNAL to LRR15) in the TLR11 family.

**Figure 8:**
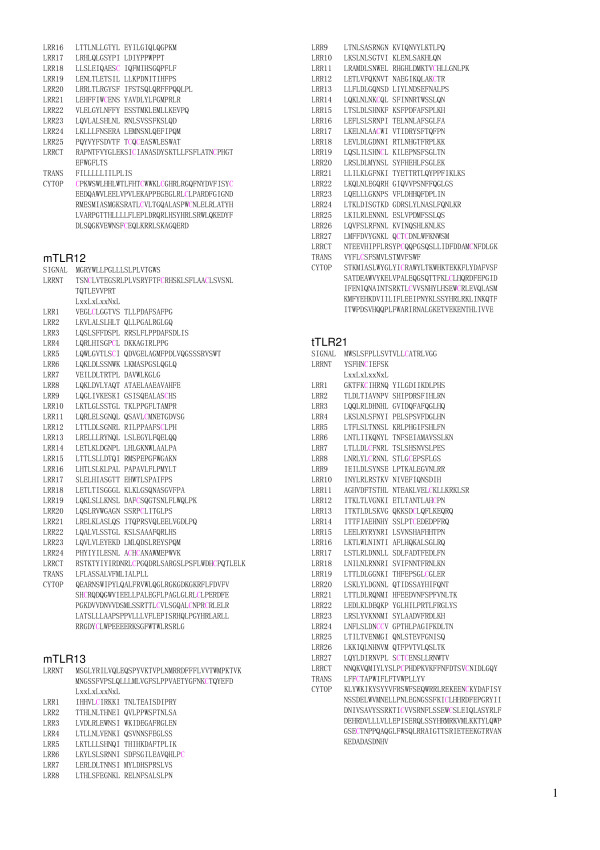
Sequence alignment of LRR domains within the six families of TLRs. This panel   continued in Figure 7 shows mtTLR11 (from LRR16 to CYTOP), mTLR12, mTLR13, and tTLR21 in the   TLR11 family.

**Figure 9:**
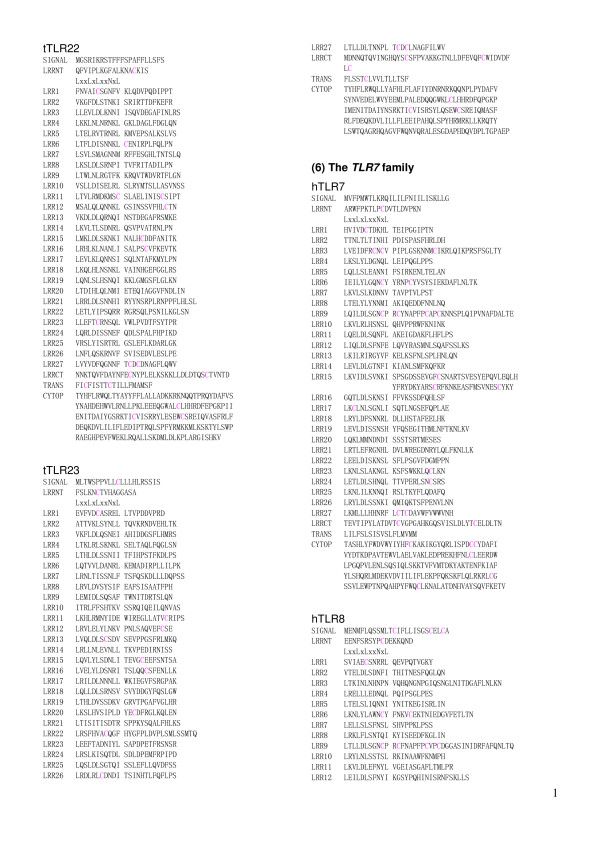
Sequence alignment of LRR domains within the six families of TLRs. This panel   continued in Figure 8 shows tTLR22 and tTLR23 in the TLR11 family, and hTLR7 and hTLR8 (from   SIGNAL to LRR12) in the TLR7 family.

**Figure 10:**
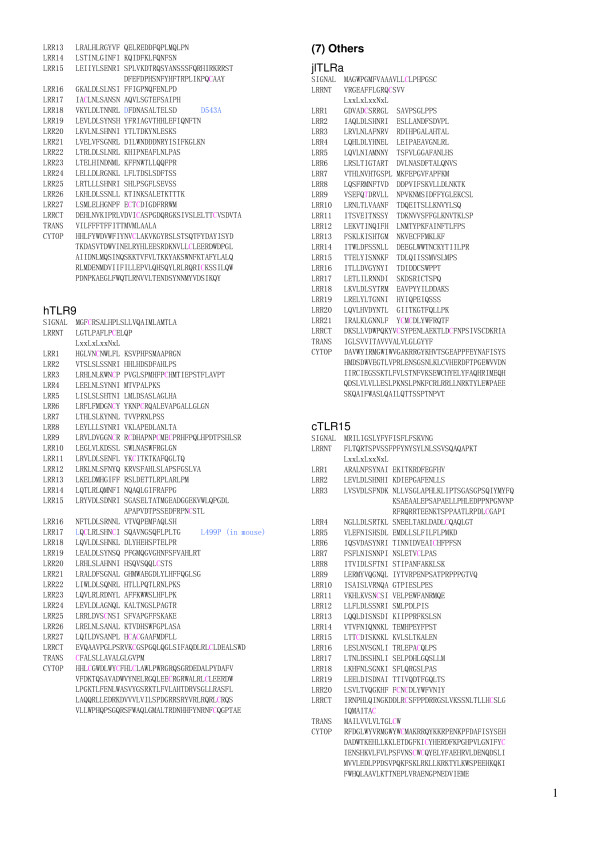
Sequence alignment of LRR domains within the six families of TLRs.  This panel   continued in Figure 9 shows hTLR8 (from LRR13 to CYTOP) and hTLR9 in the TLR7 family, and   jlTLRa and cTLR15.

The repeat number and "phasing" of LRRs in TLRs are summarized in Table 2 and Figures 5, 6, 7, 8, 9, and 10. The number of LRRs identified within TLRs range from 16 to 28; these numbers are larger than those reported in most databases. The "typical"; "***T***", LRR, LxxLxLxxNxLxxLxxxxFxxLxx, occurs most frequently followed by shorter motifs including LxxLxLxxNxLxxLPx(x)LPxx ("bacterial"; "**S**") with 19–21 residues. Moreover, all of the C-terminal LRR consists of LxxLxLxxNP(F/L)xCxCxxxx(F/L)xxxx. The TLRs contain a variety of irregular LRRs (Figures 5, 6, 7, 8, 9, and 10). The first LRR at the N-terminus (LRR1) is frequently irregular, e.g. (L/x)xx(L/A)xCxx(**L/R**)xLxxVPxxIPxx. This motif has been seen in the structures of TLR3, Slit, decorin, and biglycan, as noted. (Table 1). Methionine and tryptophan sometimes occupy positions 1, 4, 6 and 11 in the LxxLxLxxNxL motif, which are strongly hydrophobic. Moreover, as recognized in the known LRR structures, there are rare examples, **xV**xxLxLxxNxL, **P**xxLxLxxNxL and LxxLx**G**xxS/PxI. The first motif is sometimes observed in LRR7 in human TLR10, LRR8 in Takifugu rubripes TLR14, LRR4 in chicken TLR15, LRR8 in human TLR4, and LRR16 in human TLR9 (Figure 5, 6, 7, 8, 9, and 10). Furthermore, the HCS parts of a twelve residue stretch, LxxLx(L/V/M/F)xx(S/N)xx(F/M), are sometimes observed; they include LRR5 in TLR2 from pig, bovine, nilgai, and domestic water buffalo with 20 LRRs (Figures 2 and 3), LRR11 in mouse TLR4 with 23 LRRs, and LRR14 in TLR4 from pig, bovine, rabbit, and nilgai with 23 LRRs.

#### LRRs in the six major families of TLRs

There are six major families of vertebarate TLRs [4]. The *TLR1 *family consists of TLR1, TLR2, TLR6 and TLR10. This family contains 19–21 LRRs and has fewer numbers than do the other families except for Dog TLR4 [Q8SQH3] [56] and human TLR4 variant [Q5VZ17] in the *TLR4 *family. Mammalian TLR1 contains 20 LRRs (Table 2). In contrast, Takifugu rubripes TLR1 [4] has one additional LRR at the N-terminus whose sequence is **R**NYIDLSSRNLSSVPGDLPKE, that is a "bacterial" type. Mammalian and Takifugu rubripes TLR2 contains 20 LRRs. Japanese flounder TLR2 lacks one LRR that corresponds to LRR7 in the 20 LRRs [57]. Conversely, zebrafish TLR2 has one additional LRR at the N-terminus, as does Takifugu rubripes TLR1. This *TLR1 *family shows a feature that irregular LRRs mainly concentrate at the central part of the LRR domain. In the *TLR3 *family, mammalian, Takifugu rubripes and Zebrafish TLR3 contain 25 LRRs as was confirmed by the crystal structure of human TLR3 [52,53]. However, Japanese flounder TLR3 [57] contains two additional LRRs. Similarity sequence search indicates that TLRs from rainbow trout, Atlantic salmon, and goldfish are very similar to Japanese flounder TLR3. TLR4 that constitute the *TLR4 *family contains 23 LRRs. Fourteen of the 23 LRRs are similar to "typical". Seven LRRs are irregular. As seen in the *TLR1 *family, 5 of the 7 irregular LRRs are in the central part of the LRR domain. Dog TLR4 [Q8SQH3] [56] and human TLR4 variant [Q5VZ17] are shorter by about 200 amino acids at the N-terminus. These two TLRs contain only 16 LRRs. It is also predicted that dog TLR4 has no transmembrane region (Figure 7). TLR5 contains 22 LRRs. Ten of these 22 LRRs are clearly "typical". LRR15 in mammalian TLR5 is only 19 residues (Figure 7); the homolog in Takifugu rubripes and rainbow trout is 24 residues long. The *TLR11 *family contains 24–27 LRRs. Most of LRRs in mouse TLR11, TLR12, and TLR13 are "typical". The same feature is observed in Takifugu rubripes TLR21, TLR22 and TLR23. Two Japanese lamprey TLRs appear to belong to the *TLR1 *family.

**Figure 11:**
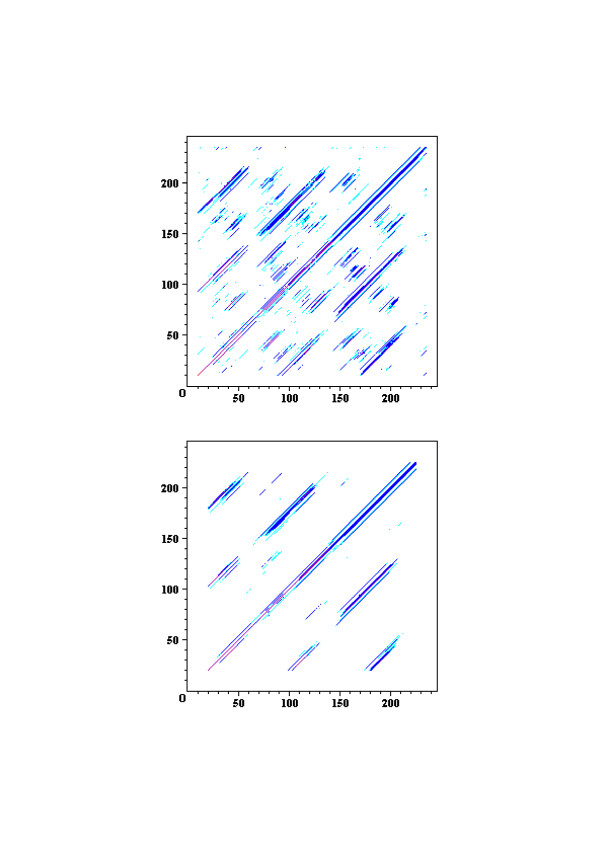
Super-repeat of LRRs in the *TLR7 *family of TLR7, TLR8 and TLR9. Forty-two superimposed, cross-dot matrices from human TLR7 [Q9NYK1], mouse TLR7 [P58682], human TLR8 [Q9NR97], mouse TLR8 [P58682], human TLR9 [Q9NR96], mouse TLR9 [Q9EQU3], and green puffer TLR [Q4S0D3] with the widow size of 21 residues and the stringency of 10 (upper) and with the widow size of 41 residues and the stringency of 20 (lower). The summed scores for the 21 ((7 × 6)/2) comparisons are represented by color. The order of higher scores is red > purple > blue > light blue. Residue 46–291, 46–291, 44–288, 44–283, 40–285, 40–285, and 23–268 of human TLR7, mouse TLR7, human TLR8, mouse TLR8, human TLR9, and green puffer TLR, respectively, were used for the cross-dot matrices. The abscissa axis and the ordinate axis are residues number.

**Figure 12:**
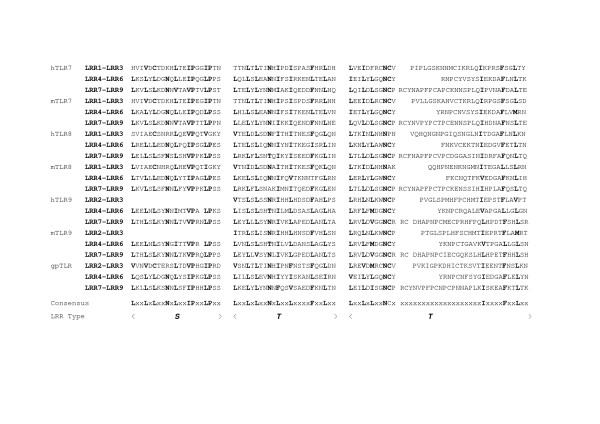
Sequence alignment of super-repeat of LRRs within TLR7, TLR and TLR9 from human and mouse and TLR from green puffer. human TLR7 [Q9NYK1]; mouse TLR7 [P58682]; human TLR8 [Q9NR97]; mouse TLR8 [P58682]; human TLR9 [Q9NR96]; mouse TLR9 [Q9EQU3]; green puffer TLR [Q4S0D3]. Abbreviations: h, human; m, mouse; gp, green puffer.

The *TLR7 *family consists of TLR7, TLR8 and TLR9 and contains 27 LRRs. Cross dot plots were computed for all of TLR7, TLR8, and TLR9 from human and mouse, and green puffer TLR. More important the super-motif is about 80 residues. Superposition of 21 ((7 × 6)/2) cross dot-plots for the seven proteins emphasize the super-repeat of LRRs at the N-terminal part of the LRR domain (Figure 11) [11]. This super-motif comes from nine LRRs from LRR1 to LRR9 in TLR7 and TLR8, and from eight LRRs from LRR2 to LRR9 in TLR9 (Figure 12). The sequence alignment reveals two types of LRR, **S **and ***T***. The type ***S ***LRR is observed in the first, fourth, and seventh of the 9 tandem LRRs. All other LRRs are type ***T***. Although the third, the sixth and the seventh LRRs is longer than the second, the fifth and the eighth LRRs, their C-terminal VS parts keep the pattern of LxxxxFxxLxx that is seen in "typical" motif. Consequently their LRRs are type ***T***. Thus, there are three super-repeats, ***STTSTTSTT***, in TLR7 and TLR8, and two and two-third super-repeats, ***_TTSTTSTT***, in TLR9. Green puffer TLR forms two horseshoe domains of LRRs. The first domain is homologous to the TLR7 family and thus contains also the super-repeat of ***STTSTTSTT ***(Figure 12). LRR15 located at the central part of the 27 LRRs consists of long amino acid sequence with 73 residues in TLR7, 64 in TLR8, and 58 in TLR9, as seen in TLR15. This long LRR motif is observed in chicken TLR15. In all the case the next LRR, LRR16, is an irregular LRR that is described by (G/N)xLxLxxNx(I/L)xxVxxxxFxxLxx is similar to "typical" motif, although position 1 in the HCS part is not occupied by leucine.

### Two cysteine clusters flanking the LRR domain

The LRRs within most of TLRs are flanked by two cysteine clusters, each of which contains two to five cysteine residues (Table 2 and Figures 5, 6, 7, 8, 9, and 10). Here the cysteine clusters on the N- and C-terminal sides of LRRs are termed LRRNT and LRRCT, respectively [58]. The N-terminal cluster usually consists of two cysteines, *Cx*_5–14_*C*, but sometimes 3, 4 or 5 cysteines. With high frequency, as noted, the last cysteine of the clusters occupies a structurally equivalent position to those of leucines in the HCS part of LRR1. The *Cx*_8_*C *motif in TLR3 and the *Cx*_10_*C *motif of TLR4 form a disulfide bond [52,59], as does the *Cx*_12_*C *motif in GPIbα [41]. The *Cx*_5–14_*C *motifs presumably form disulfide bonds. The C-terminal clusters, excepting those in three TLRs (Table 2), contain four cysteines consisting of *CxCx*_22–25_*Cx*_15–20_*C*. The spacing between the first and the second cysteine that are contained in the last LRR is the same for all the families. The other spacing appears to characterize each family. The *CxCx*_25_*Cx*_18_*C *motif in TLR3 forms two disulfide bonds between the first and the third cysteines, and between the second and the fourth cysteines [52]. Such pairs of disulfide bonds have been observed for the *CxCx*_20_*Cx*_21_*C *motif in Nogo receptor [38,39] and the *CxCx*_20_*Cx*_19_*C *motif in Slit [49]. The disulfide bond connectivity can be inferred for TLRs. The C-terminal cluster for primate TLR4 (*CxCx*_23_*Cx*_17_*C*) is different from that of other mammalian TLR4 (*CxCx*_23_*Cx*_18_*C*). Only in rainbow trout TLR5 and Takifugu rubripes TLRS5 having no TIR domain, does the C-terminal cluster consists of two cysteines. There are no N-terminal cysteine clusters in TLR1, TLR6, TLR10, TLR15, and dog TLR4. However, the N-terminal amino acid sequence flanking the LRR domain might form a capping structure.

## Discussion

### LRRs within human TLRs

The present analyses of LRRs within vertebrate TLRs indicate that there are at least two types of LRR motifs; "typical"; "***T***", LRR, LxxLxLxxNxLxxLxxxxFxxLxx and "bacterial"; "**S**", LRR, LxxLxLxxNxLxxLPx(x)LPxx. Vertebrate TLRs contain 16–28 LRRs (Table 2 and Figures 5, 6, 7, 8, 9, and 10). Bell *et al*., [60] have proposed that the ECDs of human TLRs comprised 19–25 LRRs including both "***T***" and "**S**" LRRs. Each member of human TLRs contain 1–2 times less LRRs than those identified here. Furthermore, in the *TLR1 *family (TLR1, TLR2, TLR6 and TLR10) the LRRs at the central parts are aligned differently to each other. Such a difference is also seen in TLR4, TLR5, and TLR7. The alignments of TLR3, TLR8 and TLR9 are nearly identical except the first LRR at the N-terminus of the LRR domain and the last LRR at its C-terminus.

### One or two horseshoe domains of LRRs within TLRs

The *TLR7 *family (TLR7, TLR8, TLR9 and green puffer TLR) have 27 LRRs and an additional 58–73 residues at the end of LRR15 (Figures 9, and 10). Such a long region is also observed in chicken TLR15 (Figure 10). Gibbard *et al*., [61] have considered two horseshoe domains of LRRs for human TLR8. That is, LRR15 has been separated into an LRR motif and 40 residues of undetermined structure. Most of the known LRR structures have a cap, which shields the hydrophobic core at the N- and C-terminii of LRRs. We suggest that these 40 residues function as the cap of the horseshoe structure, an intervening of hydrophobic core of LRR with a specific feature in TLRs. Thus, it can be concluded that the LRRs in vertebrate TLRs form one or two distinct horseshoe structures. Future structure determinations should resolve the question.

The *TLR1 *family (TLR1, TLR2, TLR6, and TLR10) and the *TLR4 *family share a common feature, the central part of the LRR domain has a more irregular motif compared with those at the N- and C-terminal parts. The LRR structure in the three families of *TLR1*, *TLR4 *and *TLR7 *might show a structural flexibility at the central part. Alternatively, the central part would play a key role in the function.

### The LRR arc of TLRs is flat?

The LRR arc structures can be characterized by three parameters- the inner radius of the arc (*R*), the mean rotation angle about the central axis relating one β-strand to the next (ϕ¯
 MathType@MTEF@5@5@+=feaafiart1ev1aaatCvAUfKttLearuWrP9MDH5MBPbIqV92AaeXatLxBI9gBaebbnrfifHhDYfgasaacH8akY=wiFfYdH8Gipec8Eeeu0xXdbba9frFj0=OqFfea0dXdd9vqai=hGuQ8kuc9pgc9s8qqaq=dirpe0xb9q8qiLsFr0=vr0=vr0dc8meaabaqaciaacaGaaeqabaqabeGadaaakeaacuaHvpGAgaqeaaaa@2E90@), and the tilt angle of the parallel β-strand direction per turn (*θ*_t_). A 3D circle fitting method to calculate these geometrical parameters has been developed [55]. The TLR3-LRR arc yields *R *= 26.5–26.6Å, ϕ¯
 MathType@MTEF@5@5@+=feaafiart1ev1aaatCvAUfKttLearuWrP9MDH5MBPbIqV92AaeXatLxBI9gBaebbnrfifHhDYfgasaacH8akY=wiFfYdH8Gipec8Eeeu0xXdbba9frFj0=OqFfea0dXdd9vqai=hGuQ8kuc9pgc9s8qqaq=dirpe0xb9q8qiLsFr0=vr0=vr0dc8meaabaqaciaacaGaaeqabaqabeGadaaakeaacuaHvpGAgaqeaaaa@2E90@ = 10.8–10.9° and *θ*_t _= 24.5–26.7°. The TLR3-LRR belongs to "typical" type. This *R *value is comparable to 22–36 Å for the LRR arcs in Slit, FSHr, nogo receptor, decorin, and GPIbα with "typical" LRRs [8,55,58]. In contrast, the *θ*_t _value is comparable to only those for Slit (-21°) and FSHr (-40°). Also the *θ*_t _value corresponds to 19–40° for ribonuclease inhibitor and 15° for tropomodulin with "RI-like" LRRs. That is, the TLR3-LRR arc is nearly flat. This indicates that all other TLRs except for the *TLR7 *family and TLR15 might adopt flat LRR arc.

### Super-motif of LRRs in the TLR7 family

The present analysis reveals that the *TLR7 *family consisting of TLR7, TLR8 and TLR9 and green puffer TLR contains the super-motif consisting of ***STT***. Such super motifs have been observed in various LRR proteins [8,11]. One of them is the SLRP family. The SLRP family forms five distinct subfamilies. Class I consists of biglycan, decorin, and asporin. Class II has three subclasses: lumican plus fibromodulin (IIA), PRELP plus keratocan (IIB), and osteoadherin (IIC). Class III consists of epiphycan, osteoglycin and opticin. Class IV is more distantly related and consists of chondroadherin and nyctalopin. Class V consists of podocan. Their classes except for class IV contain the super-motif. Super-motifs, ***S ***and ***T***, similar to those in SLRP are also present in asporin-like proteins from human and mouse, mouse fibromodulin-like proteins, biglycan-like proteins from sea lamprey, oligodendrocyte-myelin and glycoprotein (OMGP), the FLRT family from human, mouse and Xenopus, and human ECM2 [8,62]. Furthermore, a preliminary analysis indicates that nephrocan, a novel member of the SLRP family [63], contains an ***STT ***motif. These observations suggest strongly that "bacterial" and "typical" LRRs evolved from a common precursor.

### LRR variants in TLRs associated with diseases

A number of amino acid polymorphisms, which occur in LRRs, have been reported in TLRs. Arbour *et al*., [64] first identified two mutations of human TLR4, D299G and T399I, which were associated with diminished airway responsiveness to inhaled LPS. Since then, these two mutations have been studied for their association with various infectious and inflammatory diseases; results regarding the effects of these mutations have been inconclusive [65-71]. D299G and T399I occur in LRR11 and LRR15, respectively (Figure 7). D299G is near the convex part, while T399I is located on the loop C-terminal to the convex part. Very recently, Ohara *et al*., [72] reported that one mutation, T135A, was associated with poorly-differentiated gastric adenocarcinomas. T135A in LRR5 occur at position 9 in the HCS part (Figure 7). Such a mutation has been observed in many LRR proteins such as nyctalopin, keratocan, GPIbα, GPIbβ and GPIX, which are associated with human diseases [58]. Position 9 is generally occupied by Asn or Cy and sometimes by Thr or Ser, whose side chains form hydrogen bonds in the loop structure [58]. The T135A mutation may disrupt the hydrogen bond pattern in the loop.

Mouse TLR9 plays a role in defense against systemic mouse cytomegalovirus infection. Mice with the mutation, L499P, are highly susceptible to mouse cytomegalovirus infection and shows low levels of cytokine induction and natural killer activation on viral infection [73]. L499P is located at the short loop that connects the helical structure on the convex part (in LRR17) and the β-strand on concave part (in LRR18) (Figure 10). That is, L499P in LRR18 occur at position 1 in the HCS part. The side chain of L499 is completely buried in the LRR arc. Such a mutation is also observed in trk-A and nyctalopin, which are associated with human diseases [58]. The mutation of D543A in human TLR8 abolishes the binding of CpG DNA [61]. D543A in LRR19 occur at position 1 in the VS part. Thus, D543A is located at the edge between the convex and the concave parts of the LRR arc. The Cys-to-Ala mutations in the VS part of LRR9 (C257A, C260A, C267A, and C270A) completely abolish signaling by TLR8 [61].

Hidaka *et al*., [74] detected one mutation, F303, in human TLR3 in one of three patients with influenza-associated encephalopathy. This was a loss-of-function mutation. F303S in LRR12 is located at position 4 in the HCS part. The side chain of F303 is completely buried in the LRR arc. Two mutations, H539E and N541A, resulted in the loss of TLR3 activation and ligand binding functions [75]. These two mutations occur in LRR21.

## Conclusion

The new method of alignment proposed here rationalizes the difference in the repeat numbers of LRRs and their "phasing" within TLRs in different databases and for various species and isoforms. Moreover, the new method indicates that each of the six TLR families is characterized by their LRR motifs, their repeat numbers, and the motifs of cysteine clusters. The repeat number of LRRs is larger than those previously reported in databases. The central part in the LRR domains within the *TLR1 *family and TLR4 has more irregular motifs compared with the N- and C-terminal parts. Moreover, the *TLR7 *family contains a region with 58–73 residues in the central part of the LRR domain. The central parts are inferred to play a key role in the structure and/or function of their TLRs. The LRRs in TLRs form one or two horseshoe domains. The LRR arc of TLRs is also predicted to be nearly flat. Furthermore, the LRR super-motif in the TLR7 family suggests strongly that "bacterial" and "typical" LRRs evolved from a common precursor. The present analysis should stimulate and facilitate various experimental studies to understand the molecular mechanism of TLR-ligand interactions.

## Methods

### Known structures of LRR proteins

The structures of twenty-two different LRR proteins have been determined. They are ribonuclease inhibitor (RI) [2NBH, I1DJ, LA4Y, 1Z7X], GTPase-activating protein (RanGAP) [1YRG, 1K5D, 1K5G], tropomodulin (Tmod) [1IO0, 1PGV], S-phase kinase-associated protein 2 (Skp2) [1FQV], YopM [1G9U], four internalins, Inl-B [1D0B], Inl-H [1H6U], Inl-A [106T, 106V, 106S] and Inl-C [1XEU], spliceosomal U2A' protein [1A9N], mRNA export factor (TAP) [1FT8, 1F01], rab geranylgeranyltransferase α-subunit (RabGGTα) [1DCE, 1LTX], *Chlamydomonas *outer arm dynein light chain 1 (DLC-1) [1DS9], polygalacturonase-inhibiting protein (PGIP) [10GQ], nogo receptor/nogo-66 receptor (NgR) [10ZN, 1P9A], glycoprotein Ibα (GPIbα) [1M0Z, 1GWB, 1QYY, 1M10, 1SQ0, 1P8V, 100K, 1P9A, 1U0N], decorin [1XCD, 1XKU, 1XEC], biglycan [2FT3], Slit [1W8A], CD14 [1WWL], follicle-stimulating hormone receptor (FSHr) [1XWD], TLR3 [1ZIW, 2A0Z], and human lingo-1 [2ID5].

### Amino acid sequences

The LRRs alignments within the TLR family were made for TLR1 from four species (human [Q15399, Q5FWG5, Q6FI64, Q32MK3], mouse [Q9EPQ1], pig [Q4LDR7, Q59HI9], Takifugu rubripes [Q5H727]); TLR2 from 17 species (human [O60603], mouse [Q9QUN7, Q8K3D9, Q811T5], pig [Q59HI8, Q5DX20, Q76L24], chicken [Q9DD78 (TLR2.1), Q9DGB6 (TLR2.2)], bovine [Q95LA9], rat [Q6YGU2], dog [Q689D1], rabbit [AAM50059], goat [ABI31733], horse [AAR08196], hamster [Q9R1F8], Cynomolgus monkey [Q95M53], domestic water buffalo [Q2PZH4], Nilgai [Q2V897], Takifugu rubripes [Q5H725], zebrafish [Q6TS42], Japanese flounder [Q76CT8]); TLR3 from 9 species (human [O15455, Q4VAL2, Q504W0], mouse [Q99MB1, Q3TM31, Q499F3], bovine [Q5TJ58, Q5TJ59], rat [Q7TNI8], buffalo [Q1G1A3], Rhesus macaque [Q3BBY1], Takifugu rubripes [Q5H721], zebrafish [Q6IWL5, Q32PW5], Japanese flounder [Q76CT7, Q76CT9]; TLR4 from 17 species (human [O00206, Q5VZI7, Q5VZI8, Q5VZI9], mouse [Q9QUK6, Q5RGT4, Q8K2T5], pig [Q68Y56, Q2TNK4, Q5F4K7, Q401C7], bovine [Q9GL65, Q6WCD5, Q8SQ55], rat [Q9QX05], hamster [Q9WV82], cat [P58727], lowland gorilla [Q8SPE8], horse [Q9MYW3], Pygmy chimpanzee [Q9TTN0], olive baboon [Q9TSP2], orangutan [Q8SPE9], Nilgai [Q2V898], American bison [Q3ZD70], dog [Q8SQH3], rabbit [AAM50060]; zebrafish [Q6NV08, Q6TS41(TLR4b)]; TLR5 from 8 species (human [O60602], pig [Q59HI7], mouse [Q9JLF7], bovine [Q2LDA0], chicken [Q4ZJ82], Japanese house mouse [Q1ZZX0], Takifugu rubripes [Q5H720, Q5H716(TLRS5)], rainbow trout [Q7ZT81]); TLR6 from 5 species (human [Q9Y2C9], mouse [Q9EPW9, Q7TPC5], rat [Q6P690], pig [Q59HI6, Q76L23], bovine [Q704V6, Q706D2]; TLR7 from 4 species (human [Q9NYK1], mouse [P58681, Q548J0], dog [Q2L4T3], Takifugu rubripes [Q5H719]); TLR8 from 4 species ((human [Q9NR97, Q495P4, Q495P6, Q495P7], mouse [P58682], pig [Q865R7], Takifugu rubripes [Q5H718]); TLR9 from 12 species (human [Q9NR96[, mouse [Q9EQU3], pig [Q5I2M3, Q865R8], bovine [Q5I2M5, Q866B2], dog [Q5I2M8], cat [Q5I2M7], Japanese flounder [Q2ABQ3], horse [Q2EEY0], sheep Q5I2M4], Ma's night monkey [Q56R09], Gilthead sea bream [Q3L273, Q3L274], Takifugu rubripes [Q5H717]]; TLR10 from two species (human [Q9BXR5, Q5FWG4, Q32MI7, Q32MI8], pig [Q4LDR6, Q59HI5]); TLR11 from mouse [Q6R5P0, Q32ME8]; TLR12 from mouse [Q6QNU9]; TLR13 from mouse [Q6R5N8]; TLR14 from Takifugu rubripes [Q5H726] and zebrafish [XP_687315]; TLR15 from chicken [ABB71177], TLR21 from Takifugu rubripes [NP_001027751], TLR22 from Takifugu rubripes [Q5H723], TLR23 from Takifugu rubripes [AAW70378], and TLR from rainbow trout [Q6KCC7, Q4LBC9], Atlantic salmon [Q2A132], goldfish [Q801F9]), Japanese lamprey [Q33E92, Q33E93] and green puffer (Fragment) [Q4S0D3]).

### The prediction of secondary structure, signal peptide and membrane-spanning region in protein

The protein secondary structure prediction by SSpro4.0 [13,76,77]**a**nd Proteus [12,78] were utilized for the determination and assignments of LRRs within TLRs. Signal peptide prediction was performed by SignalP 3.0 [79,80]. The prediction of membrane-spanning regions in proteins was performed by the TMHMM Program [81,82]. The PFAM program [83] was used to detect LRRs in TLRs.

### Multiple sequence alignment, sequence similarity search and dot plot analysis

Multiple sequence alignments and sequence similarity searches were performed at Bioinformatic Center, Insitute for Chemical Research, Kyoto University [84]. Dot-matrix comparisons were performed using the Blosum90 scoring matrix. The program was made in house. Window sizes and stringencies are indicated in figure legends.

## Abbreviations

Toll-like receptor: FTLR. Toll IL-receptor: FTIR. LPS: Liopolysacchride. LRR: ECD: Ectodomain. Leucine rich repeat. HCS: Highly conserved segment of LRR. VS: Variable segment of LRR. SLRP: Small leucine-rich repeat proteoglycans: FSHr, Follicle-stimulating hormone receptor.

## Authors' contributions

NM (corresponding author) carried out the molecular genetic studies and wrote the manuscript. TT performed the dot plot analysis and contributed to the data analysis. EP performed the geometrical analysis of the known structure of TLR3. TM and MT participated in the sequence alignment. KY and YK conceived of the study, and participated in its design and coordination. All authors read and approved the final manuscript.
